# The Diagnosis of Lobar Pneumonia in Children

**Published:** 1908-11-14

**Authors:** M. G. Variot

**Affiliations:** Paris


### HOSPITAL CLINICS.

### THE DIAGNOSIS OF LOBAR PNEUMONIA IN CHILDREN.

By Dr. M. G. VARIOT, Paris.

Extracts from a Clinical Lecture: specially reported for THE HOSPITAL.

THE clinical study of lobar pneumonia in children is of great interest, because this disease is very frequent; a hundred or more cases pass through our wards every year. It may occur in quite young infants, but is more common in children over two or three years of age.

The symptoms in infantile pneumonia are often very alarming, but generally the situation appears more serious than it really is. The diagnosis, too, is sometimes rather obscure. A little girl, æt. 22 months, was brought to the hospital on account of the following symptoms: fever, loss of appetite, irritableness, and weakness. The temperature as recorded on the days following admission was: Evening, 104°, 103.7°, 104.4°; morning, between 90° and 100°. The cough was only slight and infrequent, respiration was hardly more rapid than the normal. The above symptoms would scarcely make one suspect any affection of the respiratory tract, and yet the physical examination of the chest showed distinct dulness at the apex of the left lung in the supra- and sub-spinous fossæ; tubular respiration in the left axilla with bronchophonic vocal resonance. These symptoms were distinctly circumscribed, and sufficed to make the diagnosis of lobar pneumonia in spite of the absence of any marked disturbance of respiration and irregularity of temperature.

It is relatively common to observe during the day extensive oscillations of the temperature from 98.4° in the morning to 104° or 106° in the evening, and this is especially the case towards the end of the affection. In general, the temperature falls after five or six days to normal or even below, and one might take this fall to indicate the defervescence; but this is not always so, for a few hours later it may go up again to 104° or higher. This rise might be thought to mean an extension of the disease, the development of another focus in the other lung, for instance, but in the majority of cases it is but transitory, and in 24 hours or so the temperature becomes normal.

In certain rare cases marked oscillations of temperature are present throughout the course of the disease, the difference between the morning and evening records being from 3° to 12° F. I saw, a little while ago, in consultation with two colleagues, a boy of five who had presented during five or six days oscillations of temperature as described above without it being possible to find any adequate cause. The examination of the chest was rendered extremely difficult by the restlessness of the child, but there seemed to me to be a shade of dulness under one of the clavicles. The attending physicians had, for want of a better, made the diagnosis of fever, due to gastro-intestinal infection. I suggested the possibility of a central focus of pneumonia, and two days later this was corroborated by the appearance of an area of tubular breathing on one side of the chest. In one of my wards I had a case of pneumonia with oscillating temperature in a boy two and a half years old; the physical symptoms only developed on the fifth day.

These cases show how difficult the diagnosis of acute lobar pneumonia may be, especially when a thorough physical examination is rendered anything but easy by the child being irritable and restless. In a general sort of way, it may be said that as pneumonia is easily recognised in the adult, owing to well-marked functional and physical symptoms, so, on the other hand, it is rendered difficult in children by the absence of the ordinary symptoms and the frequency of abnormal forms of the disease. In the adult we have—a sudden onset, rigor, pain in the side, cough with characteristic expectoration, even in cases of central pneumonia, dyspnœa, fever, with a fairly regular temperature of 102° or 104°, distinct physical symptoms. In children all or the greater number of these symptoms may be absent. It is not possible to know whether there has been any rigor; the pain in the side most often does not exist, for the dyspnœa and the acceleration of the respiration are much less marked than in the adult. Generally, when the patients are over five or six years old, they complain of a pain in the chest, but sometimes they localise this pain in the abdomen, especially in the right iliac fossæ, and if there happens to be at the same time vomiting and fever of sudden onset one is very apt to refer the trouble to the appendix; in fact it is not rare for our surgical colleagues to send back to the medical wards children who had been sent to them by mistake.

I remember the case of a little patient who for three days had presented repeated and almost intractable vomiting, with a temperature of 104° and marked pain in the abdomen, the walls of which were very tense. The matter vomited had been now and then bilious and even slightly stercoraceous. The attending physician had diagnosed peritonitis. When I examined the child I found a very limited focus of souffle in one axilla. It was a case of what French authors call "pneumonie emetisante," on account of the persistent reflex vomiting.

Let us return, however, to the parallel between adults and children. Dyspnœa is pretty well constant in adults, the respiratory movements are jerky, shallow, and even painful owing to the pain in the side. In children the presence of dyspnœa and the acceleration of breathing are not constant, and when these do exist they may be caused by other febrile diseases without the respiratory organs themselves being affected. In cases of bilateral pneumonia in children over ten, dyspnœa exists in a greater or lesser degree, but one cannot count upon this symptom to help the diagnosis, for it is almost always present in all generalised broncho-pulmonary affections. Cough is often absent during the first two or three days, and when present is usually slight: it is only later, when the physical signs begin to appear, that the child coughs more frequently.

The sputum is, perhaps, the most characteristic thing in the adult, and is often in itself sufficient to make a sure diagnosis. It is a well-known fact that young children do not expectorate, any mucus that is brought up being immediately swallowed again. In more than 300 cases of infantile pneumonia which I have come across I have only seen three in which there was any expectoration, and all three of these were in children between eleven and twelve years old. I wish to point out, however, in this connection that one can very often obtain sputum even in cases of quite young children by having recourse to the following little manœuvre. By means of a tongue-depressor one just touches the back of the throat, thus causing the child to cough, and one can then see a certain quantity of mucus brought up from behind the epiglottis into the pharynx, where it can be taken by a piece of cotton-wool on the end of a probe and examined microscopically for pneumococci.

Marked pyrexia is certainly the most constant symptom in acute lobar pneumonia of children. I have described already cases of irregular fever with marked oscillations which are very embarrassing and misleading for the practitioner, but these cases are the exception. As in the adult, the temperature usually goes up suddenly to 102° to 104°, and remains stationary "en plateau" during five or six days, with only feeble remissions each morning. Defervescence takes place from the fifth to the seventh day, but sometimes high temperature persists for several days with more or less marked remissions, and in this case one should examine the chest at frequent intervals, for it is often due to the development of post-pneumonic empyema. In fine, fever may be the chief symptom in infantile pneumonia, and if the area of disease is strictly limited, if it is not larger, say, than a small tangerine, if it is situated in the central part of the lung without encroaching upon the surface of the organ, the physical symptoms may then be so slight and so uncertain that diagnosis has to remain in suspense for five or six days, and only becomes possible when the defervescence appears with a sudden fall of the temperature from 104° to 98.4°, or even under.

These cases, in which the thermometer is the physician's only guide, are not so rare as one might think. In presence of a febrile state with digestive and nervous symptoms, and absence of dyspnœa or physical signs in the lungs, the idea of typhoid presents itself, or if there is at the same time repeated vomiting and an onset accompanied by convulsions followed by delirium, one necessarily thinks of meningitis. On several occasions my house physicians have presented me cases of children with the diagnosis of typhoid, and two or three days later a very distinct area of pulmonary hepatisation with its usual physical signs has developed. In these cases Widal's test is not of much use, for it often only gives results during the second week, and even later.

The diagnosis is also very embarrassing when the symptoms consist of fever with serious nervous trouble such as convulsions, delirium, or coma. Rilliet and Barthez have described a cerebral form of pneumonia which they have divided into two varieties, the eclamptic and the meningeal, according to the predominance of either convulsions or delirium and coma. It is these cases which present the maximum of difficulty, and all the more so that thorough physical examination is often practically impossible in young children who struggle and cry at the slightest attempt to touch them. However, I feel sure that many physicians have had occasion to reassure parents terrified by the thought of meningitis in children presenting serious nervous symptoms due simply to pneumonia. The following case is a very good example. A little girl of seven, of a rather nervous disposition, but who had always been in good health previously, had been attended by a specialist for acute otitis, when suddenly the temperature went up to 104°, delirium supervened, and the child became very restless and agitated. A surgeon was called, and favoured the diagnosis of meningitis, which naturally greatly frightened the parents. I saw the patient two days after the onset of the fever, and found her in a state of complete unconsciousness with rigidity of the muscles of the neck, the eyes fixed, and the pupils contracted (but not unequal); the temperature was 103.8°. As far as it was possible I made a very careful examination of the chest; auscultation was negative, but on percussion I found a slight diminution of resonance below the right clavicle; there was no dyspnœa, and the respiratory movements were not increased in rapidity. I learnt that the child had vomited several times during the morning of that same day. I made a lumbar puncture and drew off 5 or 6 grammes of clear liquid which ran out under no excess of pressure. The microscopical examination of the liquid after centrifugalization did not show the presence of any abnormal elements. Taking into consideration the results of percussion and of the examination of the cebro-spinal fluid, I reserved my opinion as to meningitis, and gave the parents hope that we might be in presence of a case of central pneumonia with marked meningeal reaction. The next day, in spite of tepid baths and the application of four leeches to the mastoid regions, the nervous symptoms increased in intensity, the agitation became extreme, the child cried out, ground its teeth, and it was absolutely impossible to administer any food. Three days later a violent attack of convulsions came on followed by coma. I was then called in again, although the parents had practically given up all hope of seeing their child recover. I found the pulse fairly strong and regular; the limbs on the left side were contracted. As the little patient was extremely restless and agitated I advised a sub-cutaneous injection of half a centigramme of morphine. This had a complete success, the patient fell rapidly into a calm sleep which lasted five or six hours, and when she awoke she began to recognise the persons round her. The next day the attending physician informed me that she had completely recovered consciousness, and that on auscultation, tubular breathing was clearly audible at the upper part of the right lung.

The diagnosis was now quite clear, and, as a matter of fact, the patient recovered in the usual time for cases of pneumonia. In dealing with cases such as this one, one must, of course, always bear in mind the possibility of tuberculous meningitis or cerebro-spinal meningitis; but my object in this lecture is simply to draw attention to the relative frequency of infantile pneumonia and the possibility of very obscure cases.

I have already mentioned the difficulty one often finds in making a thorough physical examination when dealing with a restless and unmanageable child. Whenever possible, try and quieten it for a moment, either by speaking gently to it or by giving it a sweet or a toy; then place it well on its back with a small pillow under the neck, so that the head is rather low down and the subclavicular fossæ are well projected forwards, and percuss lightly the two subclavicular regions. When there is any consolidation of the lung you will generally be able to make out more or less marked diminution of resonance on the side corresponding to the lesion, even when the latter is deep-seated. If the lesion is situated near the apex of the lung, which is very frequent in children, well-defined dulness can be heard. It sometimes happens that a well-marked tympanic note is elicited on the affected side, due to vicarious emphysema round the consolidated area; the resonance on the sound side seems then weaker than on the other, and it is not at all rare, when no other symptoms are present, to localise the trouble in the wrong lung. I cannot insist too strongly on the fact that the symptoms furnished by percussion may be detected much earlier than those given by auscultation; they may precede the latter by two or three days and even longer, and in some cases constitute the only symptoms throughout the whole course of the disease. It is practically useless to try to detect tactile vibrations in children; but bronchial souffle and bronchophony are generally very easily heard.

I was so struck, a few years ago, by the frequent absence of physical signs in infantile pneumonia that, together with my colleague, Dr. Chicotot, I tried radioscopy in the diagnosis of these cases. In cases of central pneumonia, with a well circumscribed focus of disease, radioscopy shows a distinct opacity, and the information thus obtained is all the more valuable that the physical symptoms may be either entirely absent or very doubtful. I very frequently now, when embarrassed, have recourse to this means of investigation, and do not doubt that in the future it will be used more in private practice, as it is possible to obtain a small portable apparatus, the technique of which is easily learnt. Dr. Weill, of Lyons, has recommended a method of investigation in cases of central pneumonia; he thinks there is more or less complete immobility of the chest-wall on the affected side, and uses, to render this apparent, a small paper index, placed under each clavicle. Personally, I have found this sign very inconstant; unilateral immobilisation of the chest-wall in pneumonia is not frequent, it being rather a symptom of pleurisy with effusion.

The differential diagnosis between lobar pneumonia and broncho-pneumonia is generally easy. In the latter we find dyspnœa with respirations 70, 80, and even 90 to the minute, cyanosis, and physical signs in both lungs. Percussion is generally negative, but auscultation gives numerous mucous râles, with or without souffles. Radioscopy does not give any well-defined results.

The diagnosis becomes extremely difficult when one has to do with double pneumonia, and it is the onset and course rather than the physical signs which decide one way or the other. The bacteriological examination of the sputum obtained as described above, is very useful, the presence of pneumococci rather improving the prognosis, even when there are foci of disease in both lungs.

